# Palmitoleic Acid Decreases Non-alcoholic Hepatic Steatosis and Increases Lipogenesis and Fatty Acid Oxidation in Adipose Tissue From Obese Mice

**DOI:** 10.3389/fendo.2020.537061

**Published:** 2020-09-30

**Authors:** Maysa M. Cruz, Jussara J. Simão, Roberta D. C. C. de Sá, Talita S. M. Farias, Viviane S. da Silva, Fernanda Abdala, Vitor J. Antraco, Lucia Armelin-Correa, Maria Isabel C. Alonso-Vale

**Affiliations:** ^1^Post-graduate Program in Chemical Biology – Institute of Environmental Sciences, Chemical and Pharmaceutical, Federal University of São Paulo - UNIFESP, Diadema, Brazil; ^2^Department of Biological Sciences, Institute of Environmental Sciences, Chemical and Pharmaceutical, Federal University of São Paulo - UNIFESP, Diadema, Brazil

**Keywords:** high-fat diet, n-7 fatty acid, adipocytes metabolism, hepatic triglycerides, WAT gene expression

## Abstract

We recently demonstrated that palmitoleic acid (C16:1n7), a monounsaturated fatty acid, increases the metabolic and oxidative capacity of 3T3-L1 adipocytes. Herein, the effect of 16:1n7 supplementation on metabolic parameters on white adipose tissue (WAT) and liver of obese mice induced by a high-fat diet (HFD) was addressed by analyzing metabolic (dys)function and altered genes expression in adipose tissue, as well as liver and serum biochemistry analysis. For this purpose, mice were induced to obesity for 8 weeks, and from the 5th week, they received 16:1n7 (300 mg/kg per day) or water for 30 days, by gavage. Subcutaneous inguinal (ING) and epididymal (EPI) WAT were removed for analysis of metabolic, (anti)inflammatory, adipogenic, and thermogenic genes expression by real-time reverse transcriptase–polymerase chain reaction. Additionally, metabolic activities of isolated adipocytes, such as glucose uptake, lipogenesis (triacylglycerol esterification), β-oxidation, and lipolysis in ING adipocytes, were also assessed. Despite the higher fat intake, the HFD group showed lower food intake but higher body weight, increased glucose, significant dyslipidemia, and increased liver and adipose depot mass, accompanied by liver steatosis. The 16:1n7 supplementation slowed down the body mass gain and prevented the increase of lipids in the liver. HFD+n7 animals presented increased fatty acid oxidation and lipogenesis compared to control, but no effect was observed on lipolysis and glucose uptake in ING isolated adipocytes. Besides, 16:1n7 increased the content of the mRNA encoding FABP4, but partially prevented the expression of genes encoding ATGL, HSL, perilipin, lipin, C/EBP-α, PPAR-γ, C/EBP-β, CPT1, NRF1, TFAM, PRDM16, and nitric oxide synthase 2 in ING depot from HFD group of animals. Finally, HFD increased *Mcp1* and *Tnf*α expression, and 16:1n7 promoted a more marked increase in it. In summary, the data show that palmitoleic acid promotes metabolic changes and partially prevents the increase in gene expression on adipocytes triggered by obesity, suggesting that HFD+n7 animals do not require the same magnitude of metabolic adaptation to cope with energy demand from the HFD. In the long term, the effects of 16:1n7 may be more evident and beneficial for the function/dysfunction of WAT from an obese organism, with relevant repercussions in the systemic metabolic homeostasis.

## Introduction

Obesity is the condition most often related to increased risk of developing metabolic disorders such as dyslipidemia, non-alcoholic fatty liver disease, insulin resistance and type 2 diabetes, hypertension, and cardiovascular disease, all of which contribute to higher death risk. Obesity results from the white adipose tissue (WAT) expansion due to an excessive triacylglycerol (TAG) storage triggered by a positive energy balance [e.g., during the high-fat diet (HFD)–induced obesity], which favors adipocyte hypertrophy, as well as metabolic tissue dysfunction that is closely related with a state of low-grade inflammation in WAT (1).

Increased lipogenesis and fatty acid uptake lead to TAG accumulation in adipose cells, whereas lipolysis and β-oxidation promote lipid decrease. Lipogenesis depends on lipogenic enzymes responsible for re-esterification of free fatty acids with glycerol and for *de novo* synthesis of TAG (2–4), which in turn increases the adipocyte size. On the other hand, sequential action of various lipolytic enzymes breaks down TAGs and mobilizes fatty acids (5), which may be released to the circulatory system and used as fuel by other tissues, re-esterified back to TAG, or oxidized directly inside the adipocyte (3, 4, 6, 7). Disturbances in these processes of WAT lipid metabolism, such as lipogenesis, lipolysis, and fatty acid β-oxidation, precipitates the metabolic diseases (4, 8).

Interestingly, metabolic diseases are also triggered by impaired differentiation of adipocytes in WAT (2, 9). This event, known as adipogenesis, is essential for WAT turnover and expansion in lean and obese subjects (10). It has been now accepted that a healthy WAT is characterized by the presence of smaller and more numerous adipocytes, suggestive of tissue expansion by increased adipogenesis (11).

Moreover, WAT is an endocrine organ that produces and releases numerous factors to the circulation to regulate food intake and energy expenditure, in addition to glucose and lipid metabolism, among other effects (12). Finally, WAT synthesizes and secretes palmitoleic acid (16:1n7), an omega-7 monounsaturated fatty acid that has shown beneficial metabolic effects in mice and humans (13–16). Considered a “lipokine” released by WAT, it promotes an increase in insulin sensitivity in muscle (14) and WAT (17, 18). Besides, 16:1n7 increased lipolytic and lipogenic activity, as well as oxygen consumption, fatty acid oxidation, and ATP content of 3T3-L1 white adipocytes (19). Furthermore, this fatty acid was associated with exercise-induced cardiac hypertrophy (20) and the anti-inflammatory action on macrophages (21, 22).

In an experimental model of spontaneous type 2 diabetes (KK-Ay mice), treatment for 4 weeks with 16:1n7 (300 mg/kg per day) increased insulin sensitivity and decreased the glycemic curve after glucose tolerance test (GTT) and plasma insulin concentration. Also, 16:1n7 decreased the concentration of triglycerides in plasma and reduced the gene expression of fatty acid synthase (FAS), sterol-regulatory element binding protein (SREBP-1c), stearoyl-CoA desaturase 1 (SCD1), tumor necrosis factor-α (TNF-α), and resistin in mesenteric WAT (16). Finally, in C57BL/6 mice with 12 weeks' HFD-induced obesity, the 2 weeks' treatment with 16:1n7 improved the GTT and also the insulin tolerance test (23). There is a great interest in therapies to treat obesity-associated diseases, or at least, agents that could improve some metabolic parameters triggered by this condition (such as non-alcoholic hepatic steatosis and adipose tissue dysfunction), making WAT somehow metabolically more active. Herein, we investigated the effects of palmitoleic acid on metabolic and genic parameters from WAT and liver of animals submitted to HFD-induced obesity.

## Materials and Methods

### Animals and Palmitoleic Acid Supplementation

Eight-week-old male C57BL/6 mice were maintained under controlled light–dark cycle of 12–12 h, temperature of 24 ± 1°C, and relative humidity 53 ± 2%. The mice were obtained from the Center for Development of Experimental Models (CEDEME), Federal University of São Paulo. The experimental protocol remained for 8 weeks. In the first 4 weeks (period I), mice were divided into two groups: (a) control (low fat) diet (control) and (b) HFD (obese). In the next 4 weeks (period II), the HFD group was subdivided into obese and (c) HFD supplemented with palmitoleic acid (obese+n7) groups. Control diet contains 76% carbohydrate, 15% protein, and 9% fat, and an HFD contains 26% carbohydrate, 15% protein, and 59% fat, in % kcal. Supplementation was performed by oral gavage at 300 mg/kg per day of pure palmitoleic acid (16:1n7) (Sigma, St. Louis, MO, USA) (17, 18). The control and obese groups received water by gavage at the same volume (~10 μL, according to the body weight). Gavages were carried daily between 16:00 and 17:00 h. All procedures were approved by the Ethics Committee on Animal Use of the Federal University of São Paulo (CEUA 8347020315).

### Experimental Procedure

Body weight and food intake were measured weekly, and the food and energy efficiency were calculated by the ratio of body weight gain (g) to food ingestion (g), or by ratio of body weight gain (g) to caloric intake (kcal). After 8 weeks of the experimental protocol, 10- to 12-h fasted mice were anesthetized with isoflurane and killed by cervical dislocation, and after blood was collected through puncturing the orbital plexus. Blood samples were centrifuged at 1,500 rpm for 20 min at 4°C, and serum was stored at −80°C. Liver and adipose fat depots: ING (subcutaneous), EPI (epididymal), and RP (retroperitoneal), were harvested, weighed, and processed as described below.

### Biochemical Analyses of Serum and Liver Samples

Plasma glucose, TAG, total cholesterol, low-density lipoprotein (LDL) cholesterol and high-density lipoprotein cholesterol, aspartate aminotransferase (AST), alanine aminotransferase (ALT), and gamma-glutamyltransferasase (γ-GT) levels were determined by colorimetric assays (Labtest Diagnostics, Lagoa Santa, MG, Brazil).

Liver TAG and total cholesterol content were measured in lipid extracts from previously frozen liver tissue using the same colorimetric assay kits. Fatty acids were extracted from the liver using previously described methods (24, 25). Data obtained were normalized by tissue protein concentration measured by BCA protein assay kit (Bio-Rad).

### Adipocyte Isolation

For metabolic activities studies (glucose uptake, TAG esterification, β-oxidation and lipolysis), adipocytes were isolated from subcutaneous ING fat depot, as previously described (26). A small number of adipocytes were photographed under an optical microscope (100× magnification) using a microscope camera (Moticam 1000; Motic, Richmond, British Columbia, Canada), and mean adipocyte diameter was determined by measuring 50 cells using Motic-Images Plus 2.0 software.

### Incorporation of [1-^14^C]-Palmitate Into TAG (Lipogenesis)

ING adipocytes (10^6^ cells mL^−1^) were incubated in Krebs/Ringer/phosphate buffer (pH 7.4) containing bovine serum albumin (BSA) (1%), glucose (2 mM), and palmitate (200 μM), saturated with a gas mixture of 95% O_2_ and 5% CO_2_. [1-^14^C]-palmitate, was then added to the buffer (1,850 Bq/tube) and left for 2 h at 37°C. At the end of the incubation period, the mixture was transferred to a 1.5-mL tube containing 400 μL of silicone oil and centrifuged for 30 s. The cell pellet on the top of the oil layer was transferred to polypropylene tubes containing 2.5 mL of Dole's reagent for lipid extraction. After addition of *n*-heptane (1.5 mL) and distilled water (1.5 mL), tubes were vortexed, and the mixture decanted for 5 min. An aliquot of the upper phase was collected into a scintillation vial for determination of radioactivity trapped into TAG (1450 LSC, CouterMicro-Beta, Trilux; Perkin Elmer, Waltham, MA, USA). Results are expressed as nmol of palmitate incorporated into TAG per 1 × 10^6^ cells h^−1^. A similar procedure was used in our previous study (19, 26).

### Decarboxylation of [1-^14^C]-Palmitate (Fatty Acid Oxidation)

ING adipocytes (10^6^ cells mL^−1^) were incubated in Krebs/Ringer/phosphate buffer (pH 7.4) containing BSA (1%), glucose (2 mM), and palmitate (200 μM), saturated with a gas mixture of 95% O_2_ and 5% CO_2_,. [1-^14^C]-palmitate was then added to the buffer (1,850 Bq/tube) and left for 2 h at 37°C. At the end of the incubation period, 0.2 mL of H_2_SO_4_ 8 N was injected into the tubes in order to cause the rupture of the cells and release the CO_2_ resulting from the reaction. This CO_2_ was collected and retained by adsorption on piece of Whatman filter paper (2.0 × 4.0 cm) soaked in an ethanolamine solution (0.2 mL). After 45 min of CO_2_ trapping, the filter paper was removed and transferred to scintillation vials for radioactivity counting (19). Results are expressed as nmol of CO_2_ released per 1 × 10^6^ cells h^−1^.

### 2-Deoxy-d-Glucose (2-DG) Uptake

ING adipocytes (1 × 10^6^ cells mL^−1^) were incubated with or without insulin (10 nmol L^−1^) in buffer composed of (mM): 140 NaCl, 20 HEPES, 5 KCl, 2.5 MgSO_4_, 1 CaCl_2_, and BSA 1% (pH 7.4) for 20 min at 37°C. Subsequently, 2-deoxy-D-[3H]-glucose (0.4 mmol L^−1^, 1,850 Bq per tube or well) was added, and the reaction was allowed to occur for exactly 3 min. The reaction was interrupted by adding 250 μL of ice-cold phloretin (0.3 mmol L^−1^ in Earle's salts, HEPES 10 mm, BSA 1%, and dimethyl sulfoxide 0.05%). At the end of incubation, the glucose uptake was measured as described in our previous studies (27, 28). The results are expressed in relation to the surface area (pmol/cm^2^).

### Lipolysis Measurement

The lipolysis rate was measured by quantifying the glycerol (Free Glycerol Determination Kit; Sigma) produced by the cells (1). For this, adipocytes (1 × 10^6^ cells/mL) were incubated in Krebs/Ringer/phosphate buffer (pH 7.4) containing BSA (20 mM) and glucose (5 mM) for 30 min at 37°C in the absence (basal) and presence (stimulated) of isoproterenol (2 × 10^−6^ M). The reaction was stopped on ice, and media was carefully collected for measurement of glycerol release. Results were expressed as nanomoles of glycerol per 1 × 10^6^ adipocytes.

### RNA Extraction and Quantitative Real-Time Polymerase Chain Reaction

Total RNA was extracted from ING and EPI whole adipose depots using Trizol reagent (Invitrogen Life Technologies), analyzed for quality on ratios 260/280 and 260/230 nm on NANODROP (Thermo Scientific), and reverse transcribed to cDNA using the Superscript III cDNA kit (Thermo Scientific, EUA). Gene expression was evaluated by real-time quantitative polymerase chain reaction (PCR) using a Rotor Gene (Qiagen) and SYBR Green as fluorescent dye, as previously described (29). Analysis of real-time PCR data was performed using the 2^−ΔΔC_T_^ method. Data are expressed as the ratio between the expression of the target gene and housekeeping gene (*Gapdh*). Primers used are presented in Table 1.

**Table 1:** Sense e antisense primers sequences used for real-time quantitative PCR.

**Gene**	**5^**′**^ Primer (5^**′**^-3^**′**^)-*Sense***	**3^**′**^ Primer (5^**′**^-3^**′**^)-*Antisense***
*Gapdh*	CCACCACCCTGTTGCTGTAG	CTTGGGCTACACTGAGGACC
*Tfam*	GGAATGTGGAGCGTGCTAAAA	TGCTGGAAAAACACTTCGGAATA
*Nrf1*	CGCAGCACCTTTGGAGAA	CCCGACCTGTGGAATACTTG
*Ucp1*	ACTGCCACACCTCCAGTCATT	CTTTGCCTCACTCAGGATTGG
*Pgc1-α*	ATCTACTGCCTGGGGACCTT	ATGTGTCGCCTTCTTGCTCT
*Prdm16*	CAGCACGGTGAAGCCATTC	GCGTGCATCCGCTTGTG
*Atgl*	GGTCCTCTGCATCCCTCCTT	CTGTCCTGAGGGAGATGTC
*Hsl*	GGGAGGGCCTCAGCGTTCTCACA	ATAGCACGGAGCTGGGTGAGGG
*Perilipin*	AGTGTGGGGTCCTTGGGCGT	TGGCAGCTGTGAACTGGGTGG
*Lpl*	GGCCAGATTCATCAACTGGAT	GCTCCAAGGCTGTACCCTAAG
*Fabp4*	AAGGTGAAGAGCATCATAACCCT	TCACGCCTTTCATAACACATTCC
*Lipin*	TGATGTGGTGTTCAGTGTCACT	TCGTTGACCCAGTGCAGGTA
*Dgat1*	GGCCTGCCCCATGCGTGATTAT	CCCCACTGACCTTCTTCCCTGTAGA
*Dgat2*	GAAGCTGCCCGCAGCGAAAA	TCTTGGGCGTGTTCCAGTCAA
*Fasn*	AGAGGCTTGTGCTGACTTCC	GTGGCTTCGGCGATGAGAG
*Acc1*	GAGAGGGGTCAAGTCCTTCC	ACATCCACTTCCACACACGA
*Glut-4*	CATTCCCTGGTTCATTGTGG	GAAGACGTAAGGACCCATAGC
*Leptin*	CATCTGCTGGCCTTCTCCAA	ATCCAGGCTCTCTGGCTTCTG
*Adipoq*	GCAGAGATGGCACTCCTGGA	CCCTTCAGCTCCTGTCATTCC
*C/EBP-α*	CGCAAGAGCCGAGATAAAGC	CAGTTCACGGCTCAGCTGTTC
*Ppar-γ2*	GCATCAGGCTTCCACTATGGA	AAGGCACTTCTGAAACCGACA
*Mcp1*	GCCCCACTCACCTGCTGCTACT	CCTGCTGCTGGTGATCCTCTTGT
*Tnf-α*	CCCTCACACTCAGATCATCTTCT	GCTACGACGTGGGCTACAG
*Pref-1*	GACCTGGAGAAAGGCCAGTA	AGGGAGAACCATTGATCACG
*C/EBP-β*	GCA AGA GCC GCG ACA AG	GGC TCG GGC AGC TGC TT
*Cpt1*	TGTCCAAGTATCTGGCAGTCG	CATAGCCGTCATCAGCAACC
*Nos2*	GCCACCAACAATGGCAACA	CGTACCGGATGAGCTGTGAATT
*Gpx*	CAGGAGAATGGCAAGAATGAAG	GAAGGTAAAGAGCGGGTGAG
*Cat*	GCAGATACCTGTGAACTGTC	GTAGAATGTCCGCACCTGAG
*Sod1*	AAGACTGGAAATGCTGGGAG	GGTTTGAGGGTAGCAGATGAG
*Sod2*	TGCTCTAATCAGGACCCATTG	CATTCTCCCAGTTGATTACATTCC

*Gapdh, Glyceraldehyde-3-phosphate Dehydrogenase; Tfam, Transcription Factor A, Mitochondrial; Nrf1, Nuclear Respiratory Factor 1; Ucp1, Uncoupler protein 1; Pgc1-α: Peroxisome Proliferator-Activated Receptor Gamma Coativator 1-alpha; Prdm16, PR-domain containing 16; Atgl, adipocyte triacylglycerol lipase; Hsl, hormone sensitive lipase; Lpl, Lipoprotein lipase; Fabp4, Fatty Acid Binding Protein 4; Dgat1, Diacylglycerol acyltransferase 1; Dgat2, Diacylglycerol acyltransferase 1; Fasn, fatty acid sinthase; Acc1, Acetyl-CoA carboxylase; Glut-4, glucose transporter 4; Adipoq, adiponectin; C/EBP-α, CCAAT/enhancer-binding protein alpha; Ppar-γ2, Peroxisome proliferator-activated receptor gamma; Mcp1, monocyte chemoattractant-1; Tnf-α, Tumor necrosis factor alpha; Pref-1, preadipocyte factor 1; C/EBP-β, CCAAT/enhancer-binding protein beta; Cpt1, carnitine palmitoyltransferase 1; Nos2, nitric oxide synthase 2; Gpx, glutathione peroxidase; Cat, Catalase; Sod1, superoxide dismutase 1; Sod2, superoxide dismutase 2*.

### Statistical Analysis

Data are presented as mean ± SEM. One-way analysis of variance (ANOVA) followed by Tukey posttest was used for the comparison between groups. GraphPad Prism 8.4.4 version (GraphPad Software, Inc., San Diego, CA, USA) was used for analysis. The level of significance was set at *p* < 0.05.

## Results

### Palmitoleic Acid Partially Prevented Food and Energy Efficiency Increase and the Body Mass Gain of Animals Submitted to Obesity by HFD

To characterize the obesity model, we evaluated the animals' food intake and food and energy efficiency. The HFD promoted a significant decrease in food intake (by 50%; *P* < 0.05), as well as increased both food and energy efficiency (2,6- and 1,5-fold, respectively; *P* < 0.05), and 16:1n7 supplementation partially prevented the increase in food, as well as energy efficiency. Besides, palmitoleic acid did not affect food intake, because there was no difference between groups of obese animals. We also measured glucose, triglycerides, and total cholesterol and fractions plasma concentrations. HFD promoted an increase in these parameters in the plasma, but palmitoleic acid did not affect these parameters (Table 2).

**Table 2:** Dietary intake, food and energy efficiency, fasting plasma glucose, triglycerides, total and fractions cholesterol, and adipose tissues mass.

	**Control**	**Obese**	**Obese+n7**
Food ingestion (g/day/animal)–Period I	4.40 ± 0.142	2.35 ± 0.037^*^	-
Food ingestion (g/day/animal)–Period II	4.18 ± 0.10	2.10 ± 0.01^*^	2.04 ± 0.02^*^
Food efficiency–Period I	0.22 ± 0.003	1.35 ± 0.035^*^	-
Food efficiency–Period II	0.164 ± 0.002	0.597 ± 0.004^*^	0.471 ± 0.004^*^^#^
Energy efficiency–Period I	0.059 ± 0.001	0.25 ± 0.007^*^	-
Energy efficiency–Period II	0.043 ± 0.001	0.111 ± 0.001^*^	0.088 ± 0.001^*^^#^
Glucose (mg/dL)	205.28 ± 15.50	285.48 ± 36.03	282.77 ± 32.19
Triglycerides (mg/dL)	64.19 ± 3.37	82.88 ± 4.25^*^	83.67 ± 3.72^*^
Total cholesterol (mg/dL)	152.78 ± 4.83	200.56 ± 9.92^*^	193.90 ± 6.57^*^
HDL cholesterol (mg/dL)	74.67 ± 4.94	89.89 ± 3.09^*^	92.97 ± 3.70^*^
LDL cholesterol (mg/dL)	59.58 ± 4.72	90.49 ± 9.88^*^	90.85 ± 7.68^*^
VLDL cholesterol (mg/dL)	12.25 ± 0.79	16.56 ± 0.85^*^	16.73 ± 0.74^*^
Subcutaneous (ING) mass (g)	0.25 ± 0.05	1.12 ± 0.17^*^	0.98 ± 0.08^*^
ING adipocytes diameter (μm)	60.21 ± 1.89	81,90 ± 2.61^*^	84.08 ± 1.97^*^
Epididymal mass (g)	0.55 ± 0.04	1.60 ± 0.18^*^	1.59 ± 0.11^*^
Retroperitoneal mass (g)	0.12 ± 0.01	0.49 ± 0.04^*^	0.52 ± 0.04^*^
Brown mass (g)	0.07 ± 0.01	0.09 ± 0.01	0.09 ± 0.01

*Dietary intake, energy and dietary efficiency per period. In the period I (week 1–4), the animals received control or high fat diet (Obese group). In the period II (week 4–8), the diets were maintained, and the animals received water (Control and Obese groups) or palmitoleic acid (Obese+n7 group) by oral gavage. Results were expressed as Mean ± SEM (n = 36). Data were analyzed by one way ANOVA*.

* *p < 0.05 vs. Control*,

# *p < 0.05 vs. Obese*.

**Figure 1:**
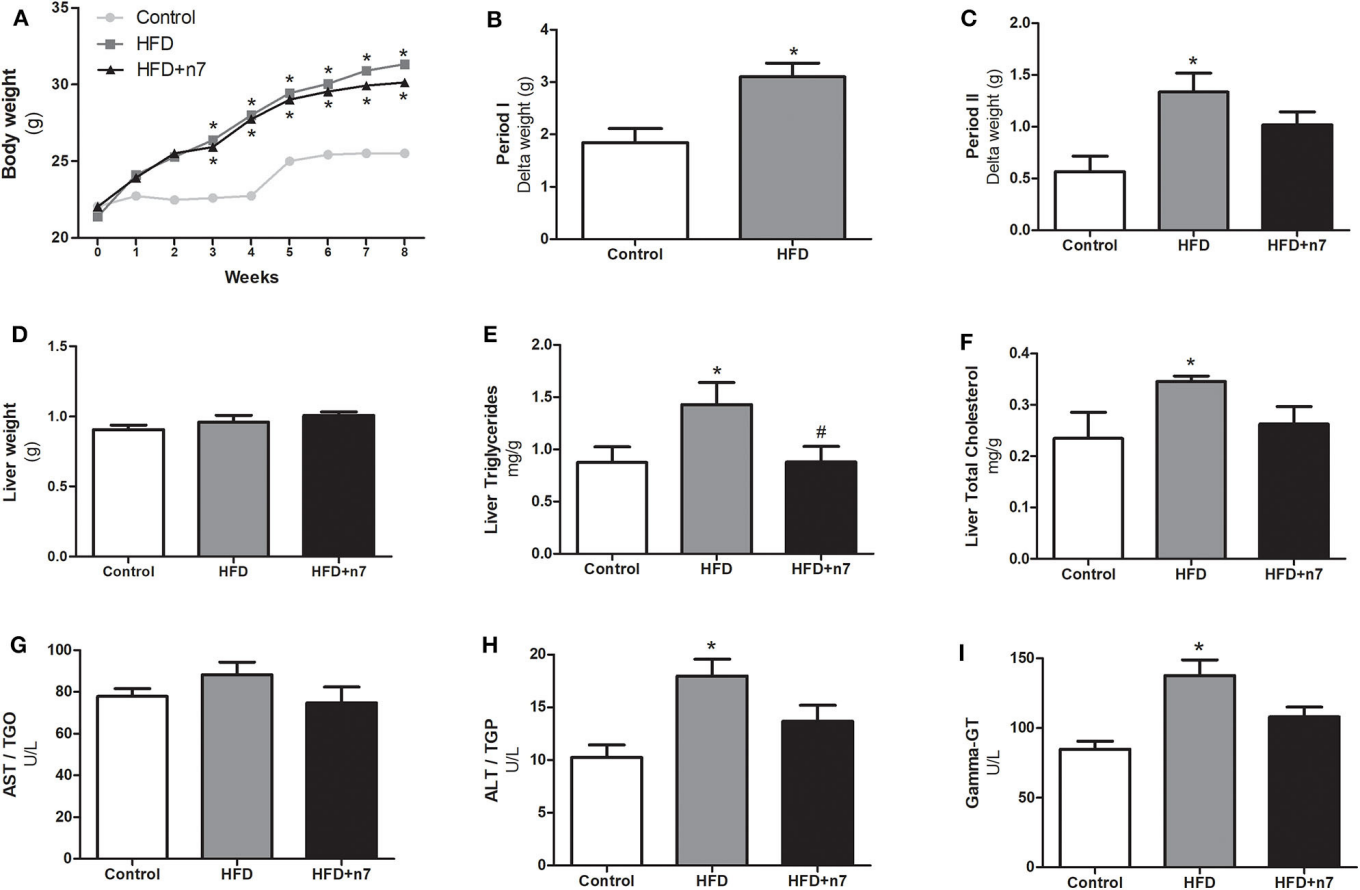
Weight gain curve **(A)**, Body mass gain in period I **(B)** and II **(C)**, liver mass **(D)**, hepatic triglyceride in mg/g of liver weight **(E)**, total hepatic cholesterol in mg/g of liver weight **(F)**, AST **(G)**, ALT **(H)**, and γ-GT **(I)** levels in U/L in plasma. In period I (weeks 1–4), the animals received control or high-fat diet (HFD). In period II (weeks 4–8), the diets were maintained, and the animals received either water (control and HFD groups) or palmitoleic acid (HFD+n7 group) by oral gavage. Results were expressed as mean ± SEM (*n* = 12). Data were analyzed by one-way ANOVA. *p < 0.05 vs. control, #*p* < 0.05 vs. obese.

The weight gain curve of the animals is shown in Figure 1A. We observed that the animals from the HFD group had an increase in body mass when compared to the control group. To analyze whether palmitoleic acid had any effect on the weight gain of obese animals, we analyzed the body mass gain during period II (weeks 4–8) of the experimental protocol, as well as the mass of the animals' fat pads. The HFD promoted an increase in body mass gain when compared to the control group in period I (by 70%; Figure 1B) and II (by 130%; Figure 1C), and palmitoleic acid was able to partially prevent this effect (decrease of 25% compared to HFD; *P* < 0.05; Figure 1C), even without exerting any effect on the mass of the subcutaneous (ING), epididymal, retroperitoneal, and brown fat pads or on the cell diameter of ING adipocytes (Table 2).

### Palmitoleic Acid Prevented Hepatic Steatosis in Obese Mice

To assess the effect of the HFD as well as palmitoleic acid in liver, the following parameters were analyzed: liver mass, triglyceride, and total cholesterol. Also, the AST, ALT, and γ-GT enzyme plasma concentrations were determined. HFD had no effect on the mass of liver (Figure 1D), but significantly increased: liver triglycerides and total cholesterol concentration and ALT and γ-GT plasma concentrations (87, 57, 80, and 63%, respectively; *P* < 0.05; Figures 1E–G,I). Palmitoleic acid was able to bring liver triglycerides to the same concentrations as in control animals (Figure 1E) and promoted a partial decrease in the other parameters (Figures 1F,H,I).

### Palmitoleic Acid Increased TAG Esterification and Oxidation of Fatty Acids in Subcutaneous (ING) WAT of Obese Mice

**Figure 2:**
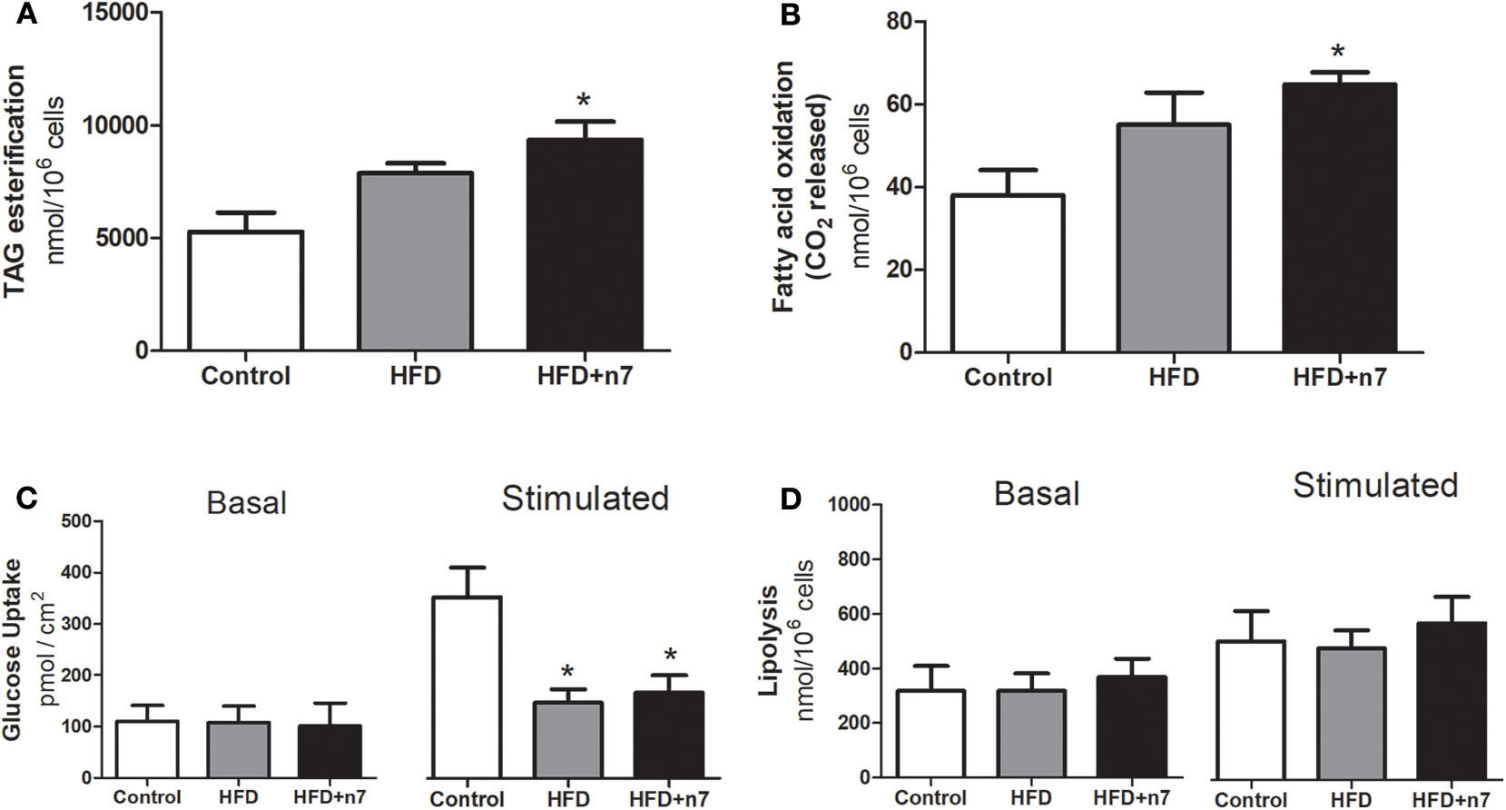
Metabolic activities in isolated adipocytes from ING fat depot. Lipogenesis/TAG esterification in nmol/10^6^ cells **(A)**, fatty acid oxidation in nmol/10^6^ cells **(B)**, basal and insulin-stimulated glucose uptake in pmol/cm^2^ **(C)**, basal and stimulated (by isoproterenol) lipolysis in nmol/10^6^ cells **(D)**. Results were expressed as Mean ± SEM (*n* = 6). Data were analyzed by one-way ANOVA. *p < 0.05 vs. control.

Taking in account that palmitoleic acid partially prevented the food and energy efficiency, as well as the body mass gain, we next evaluated the effects of palmitoleic acid on the adipose tissue metabolism in animals consuming a HFD. Palmitoleic acid promoted a significant increase in TAG esterification (by 80%; *P* < 0.05; Figure 2A) and fatty acid oxidation (by 70%; *P* < 0.05; Figure 2B).

HFD induced a significant reduction in maximally insulin-stimulated glucose uptake in obese animals compared with control animals (58%; Figure 2C), and this effect was not prevented by 16:1n7 supplementation.

Finally, ING adipocytes were evaluated for lipolysis activity (basal and stimulated by isoproterenol), but no statistical difference was observed between the groups (Figure 2D).

### Palmitoleic Acid Partially Modulates the Expression of Genes Involved in Lipogenesis (Uptake and Fatty Acid Esterification), Lipolysis, and Fatty Acid Oxidation

**Figure 3:**
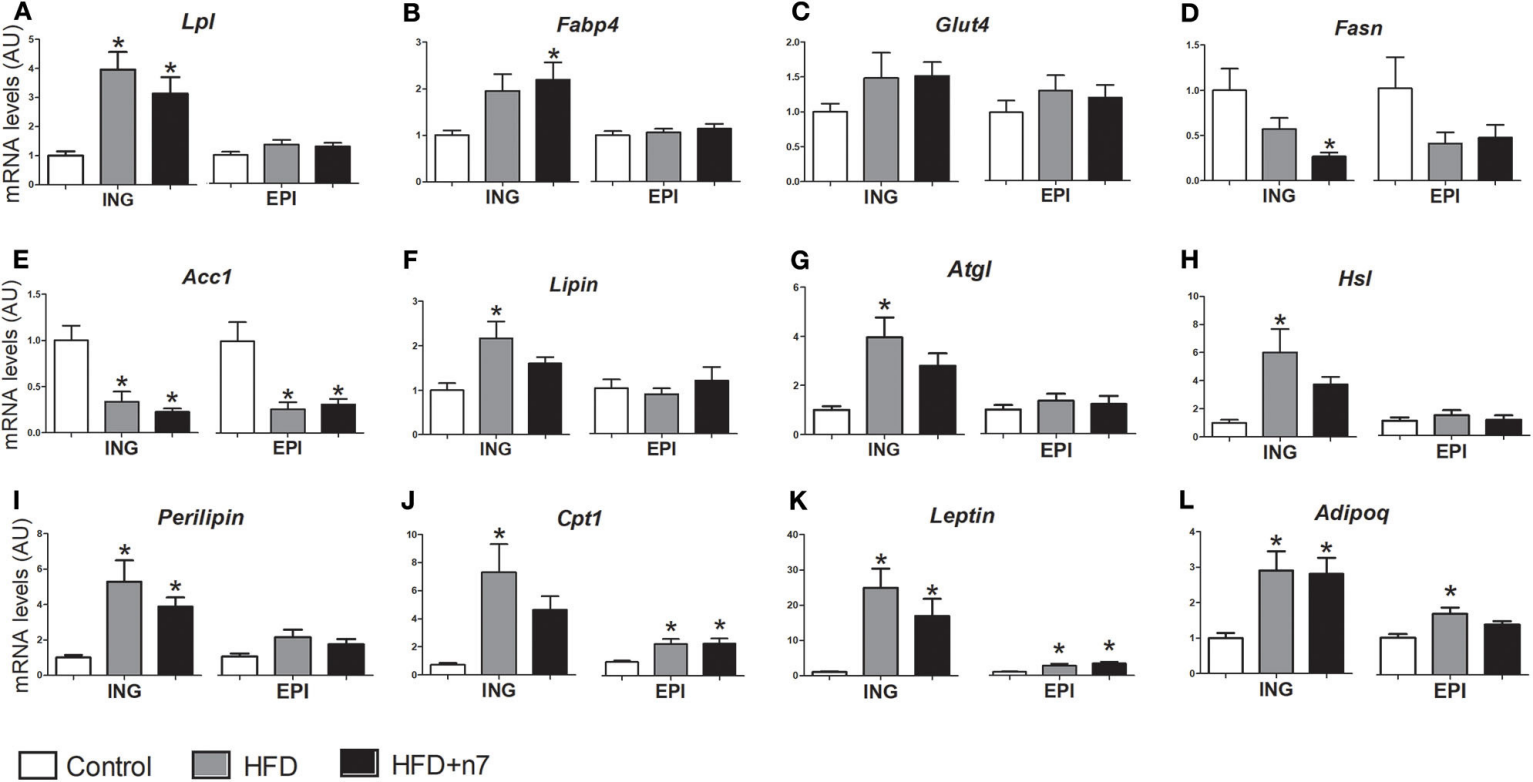
mRNA levels of *Lpl* **(A)**, *Fabp4* **(B)**, *Glut4* **(C)**, *Fasn* **(D)**, *Acc1* **(E)**, *lipin* **(F)**, *Atgl* **(G)**, *Hsl* **(H)**, *perilipin* **(I)**, *Cpt1* **(J)**, *leptin* **(K)**, *Adipoq* **(L)** expressed by isolated adipocytes from ING and EPI fat depots in arbitrary units (AU), normalized by *Gapdh*. Results were expressed as mean ± SEM (*n* = 12). Data were analyzed by one-way ANOVA. *p < 0.05 vs. control.

The expression of genes encoding important proteins and enzymes involved in metabolic activities such as glucose and fatty acids uptake (*Lpl, Fabp4*, and *Glut-4*), *de novo* synthesis and esterification of fatty acids—lipogenesis (*Fasn, Acc1, lipin, Dgat1*, and *Dgat2*), lipolysis (*Atgl, Hsl*, and *perilipin*)—and fatty acid oxidation (*Cpt1*), as well as endocrine function (*leptin* and *Adipoq*), were analyzed in subcutaneous ING and visceral EPI fat depots and are shown in Figure 3.

HFD increased the expression of *Lpl* and *perilipin* (by 3- and 4.3-fold, respectively; *P* < 0.05; Figures 3A,K) in ING depot and decreased the expression of *Acc1* (by 70%; Figure 3E) in ING and EPI depots. Palmitoleic acid positively modulated *Fabp4* expression (by 120%; Figure 3B) and decreased *Fasn* expression (74%; Figure 3D) only in ING WAT. *Glut4* gene expression was not modulated (Figure 3C).

The HFD also increased *lipin, Dgat1, Dgat2, Atgl, Hsl*, and *Cpt1* expression in ING depot, but palmitoleic acid treatment partially prevented (Figures 3F,I,J,L) or completely abolished (Figures 3G,H) this effect. These genes expression were not modulated by HFD or palmitoleic acid in ING fat depot, except by *Cpt1*, whose expression increased (2-fold; Figure 3J) in both HFD and HFD+n7 groups.

Finally, HFD consumption caused an increase in *leptin* (Figure 3K) and *Adipoq* (Figure 3L) gene expression in both ING and EPI depots, which was not altered by treatment with palmitoleic acid.

### Palmitoleic Acid Prevents Effects Promoted by the HFD on the Expression of Adipogenic and Mitochondrial Genes

**Figure 4:**
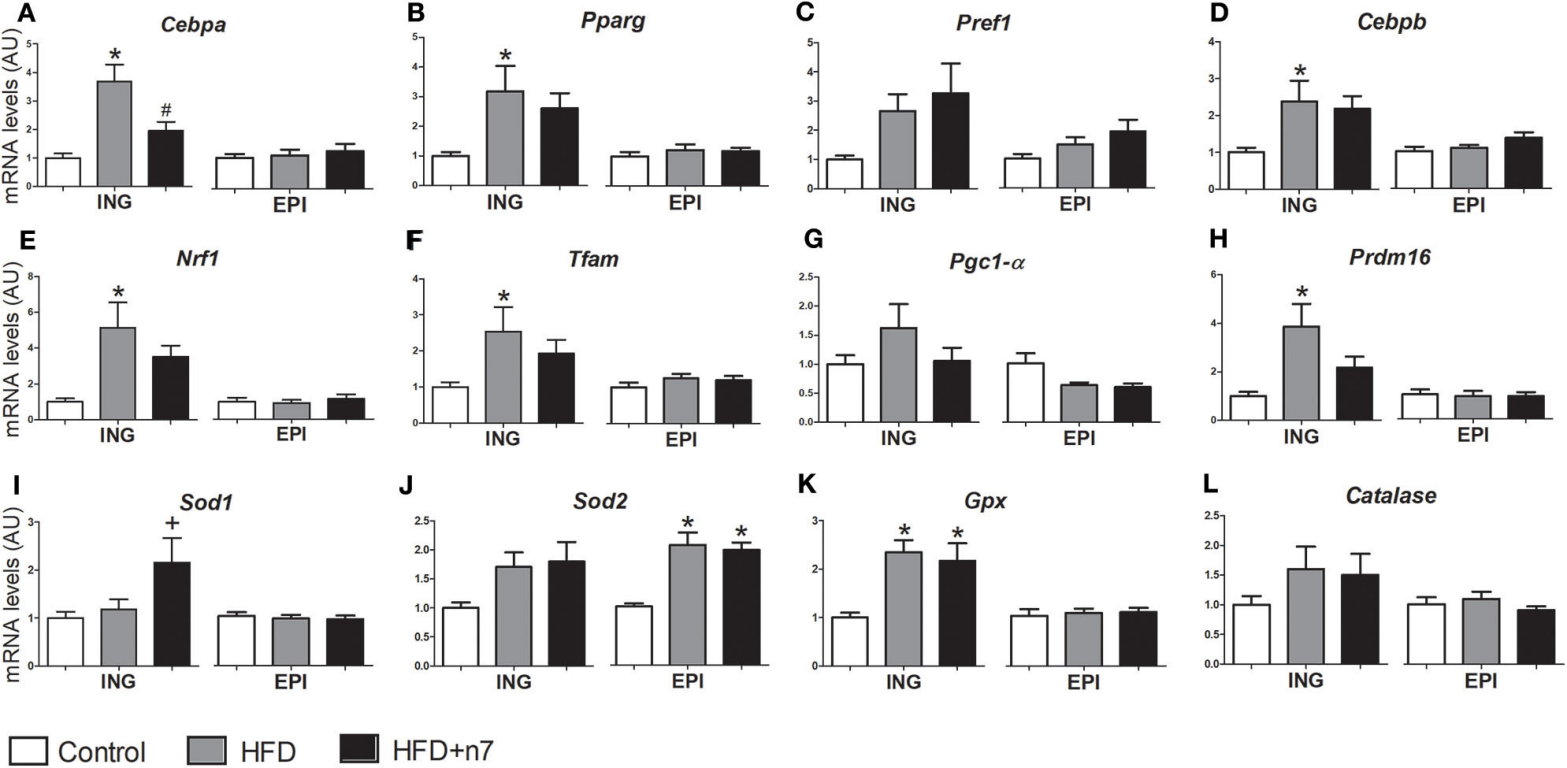
mRNA levels of *Cebpa* **(A)**, *Pparg* **(B)**, *Pref1* **(C)**, *Cebpb* **(D)**, *Nrf1* **(E)**, *Tfam* **(F)**, *Pgc1-*α **(G)**, *Prdm16* **(H)**, *Sod1* **(I)**, *Sod2* **(J)**, *Gpx* **(K)**, and *Catalase* **(L)** expressed by isolated adipocytes from ING and EPI fat depots in arbitrary units (AU), normalized by *Gapdh*. Results were expressed as mean ± SEM (*n* = 12). Data were analyzed by one-way ANOVA. *p < 0.05 vs. control, #*p* < 0.05 vs. obese, +*p* = 0.05 vs. all groups.

Regarding the effects of the HFD and palmitoleic acid on the expression of genes involved in adipogenesis (*Cebpa, Pparg, Pref1*, and *Cebpb*), mitochondrial biogenesis (*Nrf1, Tfam, Pgc1alfa*, and *Prdm16*), and antioxidant function (*Sod1, Sod2, Gpx*, and *Catalase*), the data are illustrated in Figure 4.

In ING fat depots, the expression of *Cebpa* (Figure 4A), *Pparg* (Figure 4B), *Cebpb* (Figure 4D), *Nrf1* (Figure 4E), *Tfam* (Figure 4F), *Prdm16* (Figure 4H), and *Gpx* (Figure 4K) was higher in the HFD group compared to control. Palmitoleic acid partially prevented all these increases, except for *Cebpa* expression that was reduced to control levels, and *Gpx*, which was not modulated by 16:1n7. Also, palmitoleic acid significantly increased *Sod1* gene expression (by 80%; Figure 4I). There was no significant difference in *Pref1* (Figure 4C), *Pgc1-*α (Figure 4G), *Sod2* (Figure 4J), and *catalase* (Figure 4L) expression levels. On the other hand, in EPI fat depots, the expression of adipogenic and mitochondrial genes was not modulated by our treatment, except by an increase in *Sod2* gene expression (2-fold; Figure 4J) in both HFD and HFD+n7 groups.

### Palmitoleic Acid Modulated the Expression of Genes Related to Inflammation

We also investigated, by real-time quantitative PCR, the effect of HFD, associated or not with 16:1n7, on the expression of genes related to inflammation (*Mcp1, Tnf-*α, *Nos2)* in ING EPI fat depots.

**Figure 5:**
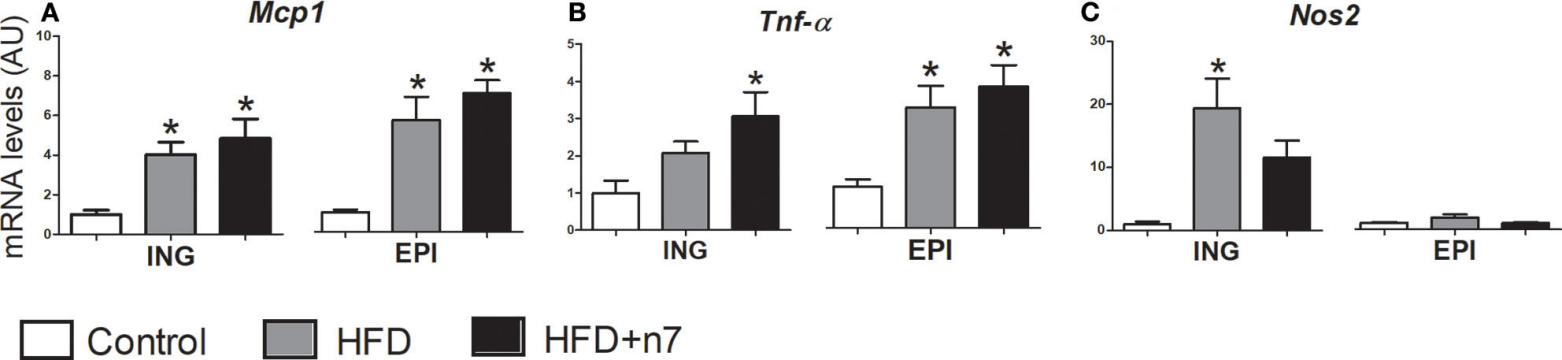
mRNA levels of *Mcp1* **(A)**, *Tnf-*α **(B)**, and *Nos2* **(C)** expressed by isolated adipocytes from ING and EPI fat depots in arbitrary units (AU), normalized by *Gapdh*. Results were expressed as mean ± SEM (*n* = 12). Data were analyzed by one-way ANOVA. *p < 0.05 vs. control.

HFD consumption caused an increase in *Mcp1* (Figure 5A) in both ING and EPI fat depots and an increase in *Tnf-*α (Figure 5B) in EPI depot. Palmitoleic acid treatment did not reverse these effects. On the other hand, in ING fat depot 16:1n7 significantly increased the expression of *Tnf-*α (2-fold; Figure 5B) and partially prevented the increase in *Nos2* expression (Figure 5C) triggered by the HFD.

## Discussion

In the present work, we have tested the effects of palmitoleic acid in C57BL/6 mice fed a HFD for 8 weeks. We show that palmitoleic acid promotes metabolic and transcriptional changes in adipose tissue, improving mice hepatic parameters. Interestingly, palmitoleic acid prevented an increase in the expression of Cebpa and Pparg transcription factors, which were induced by HFD in subcutaneous ING adipocytes. The expression of several CEBPα and PPARγ target genes, such as *Atgl, Hsl*, and *perilipin* (lipolysis-related genes); *Lpl, lipin, Dgat1*, and *Dgat2* (fatty acid uptake and lipogenesis-related genes); *leptin* and *Nos2* (obesity inflammation-related genes); and *Cpt1, Nrf1, Tfam*, and *Prdm16* (fatty acid oxidation and biogenesis-related genes) were also partially reversed in the ING fat depots of animals treated with palmitoleic acid. Therefore, palmitoleic acid seems to mitigate the impacts triggered by HFD. Our results suggest that WAT from animals subjected to HFD and palmitoleic acid gavage does not have the same adaptation challenge inflicted by HFD as that from animals subjected only to HFD and water gavage.

The challenge inflicted in animals fed an HFD led to a disruption of metabolic and energy homeostasis, with drastic repercussions in body mass and plasma lipid profile (increased triglycerides, total cholesterol, and LDL cholesterol). Treatment of HFD-induced obese animals for 4 weeks with palmitoleic acid could not reverse these features, although it prevented (even if very partially) the body mass gain. This finding is reinforced by the fact that palmitoleic acid partially prevented an increase in food and energy efficiency.

In agreement with previous findings in obese animals with hepatic steatosis (23), palmitoleic acid was not able to reduce the increment in WAT induced by HFD. We hypothesize that because palmitoleic acid is a nutrient it may take longer than 4 weeks to exert significant phenotypic alterations in WAT. Nevertheless, it exerts subtle effects that may bring important long-term repercussions.

The analysis of ING adipocytes' size (average cellular diameter) shows that they are hypertrophic. It is well-established in the literature that there is an important increase in cellular diameter in obesity, especially in pathological obesity (27, 30). We show that the treatment of obese animals with palmitoleic acid for 4 weeks could not attenuate adipocytes' hypertrophy.

We show that the 4 weeks' treatment with palmitoleic acid could prevent the increase in hepatic triglycerides and total cholesterol content and hepatic enzymes (ALT and γ-GT) promoted by HFD, even though the liver mass was not different among the groups. Previous work shows that the treatment with palmitoleic acid for only 15 days did not exert hepatic effects (23). Yang et al. (16) showed that palmitoleic acid supplementation promoted a reduction of hepatic triglycerides content in a model of diabetic genetically modified mice. It has been also shown that 16:1n7 improves hepatic steatosis in FABP knockout mice (aP2-mal1^−/−^) (14). Taken together, these findings strongly suggest that palmitoleic acid is a candidate for the prevention of liver steatosis caused by HFD.

Interestingly, a comparison of the results on fatty acid oxidation and TAG esterification in subcutaneous WAT of animals from both groups revealed that only HFD+n7 animals had an increase in both parameters. We have previously shown, in 3T3-L1 adipocytes, that palmitoleic acid increases not only lipogenesis and palmitate oxidation (19) but also lipolysis and glucose uptake and oxidation (17–19). Herein, our experimental model associates palmitoleic acid treatment to an HFD-induced obesity condition. Our results suggest that 16:1n7 promotes a persistent increase in lipogenesis (fatty incorporation into TAG) and fatty acid oxidation in WAT adipocytes. In agreement with these data, when we compared HFD group with HFD+n7, the former presented a slightly higher expression of *Lpl* gene in ING fat depots. *Lpl* encodes an enzyme (lipoprotein lipase) that hydrolyzes TAG molecules found in lipoproteins such as chylomicrons and very low-density lipoproteins. Palmitoleic acid also partially prevented the increase in expression of *lipin* but significantly hampered the increase in *Dgat1*, and *Dgat2* gene expression, which encode enzymes related to fatty acid esterification for TAG synthesis. On the other hand, palmitoleic acid promoted a significant increase in Fabp4 expression, a protein that dictates the partitioning of lipids inside adipocytes. Therefore, one hypothesis is that while HFD leads to higher plasma and liver lipid concentration, palmitoleic acid promotes an increase in fatty acid uptake by ING adipocytes, incorporating that as neutral lipids or phospholipids, among other possibilities in the cell metabolism, such as β-oxidation.

It is well-known that hypercaloric (glucose) diet increases *de novo* lipogenesis (31–34), leading to an increase of circulating palmitoleic acid. Therefore, palmitoleic acid is considered a marker of *de novo* lipogenesis (14). It has also been described that *de novo* lipogenesis reduces the effects of HFD (27, 35, 36). Corroborating the literature, we found that Fasn and Acc1 gene expression was reduced in the HFD group, but these reductions were more significant in the HFD+n7 group for both ING and EPI fat depots. These data reinforce our previous findings showing that 16:1n7 reduces *de novo* lipogenesis (17). Even though there is a reduction in *de novo* synthesis of fatty acids, animals fed an HFD presented a significant increase in lipogenesis by an increase in TAG esterification.

Glucose uptake was also analyzed in subcutaneous ING adipocytes in basal and insulin-stimulated states. We did not detect any differences in basal glucose uptake among control, HFD, and HFD+n7 groups, although the HFD has significantly reduced the insulin-stimulated glucose uptake in both HFD and HFD+n7 groups. Although it was described by other researchers that 16:1n7 increases muscle glucose uptake (14), and we have previously demonstrated this increase in eutrophic mice epididymal WAT and 3T3-L1 adipocytes (17), we could not detect 16:1n7 effects in glucose uptake and GLUT4 mRNA levels in ING fat depot of HFD-fed mice. On the other side, mice fed an HFD for 12 weeks and treated for 2 weeks with palmitoleic acid after the 10th week presented lower glucose tolerance and higher insulin sensitivity (23).

Concerning the lipolysis, the results showed that HFD did not modulate this pathway in subcutaneous WAT. Also, palmitoleic acid has not affected lipolysis in obese animals, although we have previously demonstrated that 16:1n7 treatment increases lipolysis in epididymal white adipocytes from eutrophic mice and in 3T3-L1 cell line (18, 19). Even though palmitoleic acid did not (significantly) decrease the high expression of Atgl, Hsl, and perilipin (lipolysis related genes) observed in the HFD group, there was a clear tendency to reduce it, although no difference was detected in the lipolytic assay in the ING adipocytes. It is important to emphasize that we did not perform the measures in a visceral adipose depot but in a subcutaneous depot (ING), whose expansion is believed to protect against pathologic visceral adipose expansion in obesity. Different from visceral adipocytes, whose hypertrophy is positively correlated with metabolic diseases and undergo drastic changes in size due to diet (6–10 times more lipolytic), ING adipocytes are less affected by HFD (27).

In agreement with our findings, it was demonstrated that there is an increase in perilipin gene expression in obesity (37). It was described that depending on perilipin activation status, it can act protecting and delimitating the lipid droplet or facilitating TAG hydrolysis (38).

The HFD promoted a substantial increase in the expression of Cpt1, Nrf1, Tfam, and Prdm16 in ING (but not in EPI) fat depot, which are genes related to fatty acid oxidation, mitochondrial function, and biogenesis (29, 39, 40). The treatment with palmitoleic acid partially prevented these effects in the subcutaneous WAT depot. These findings lead us to hypothesize that mitochondria could undergo a metabolic adaptation, increasing adipocytes' mitochondrial activity, trying to deal in this early stage of obesity, with the high supply of fatty acids from HFD. Obese individuals have higher SOD2, PPARα, and PGC1β protein expression than eutrophic individuals (41). Considering the increase in fatty acid oxidation in HFD+n7 animals, it seems that even though these animals received the same diet as the HFD group, they do not need to adapt in the same proportion. Warfel et al. (42) have not detected any difference in *Cpt1* gene expression in C57BL/6 subcutaneous WAT, although their obesity experimental model lasted 40 weeks. Confirming the failure in mitochondrial biogenesis with obesity progression, ob/ob mice showed reduced expression in *Nrf1, Tfam*, and *Pgc-1*α (43). PRDM16 induces Pgc-1α and Ucp1 expression and is a critical molecular unit that controls browning differentiation (44). Besides acting on the browning process, PRDM16 increases mitochondrial biogenesis, uncoupling, as well as oxygen consumption in WAT (45). Mitochondria have a crucial role in controlling the energetic balance, ATP synthesis, energy expenditure, and energetic support during adipogenesis, among several other functions. Excess of energy substrates leads to mitochondrial dysfunction, harming glucose, and lipid cell metabolism. Adipocytes help to maintain the proper balance between energy storage and expenditure. This balance requires a preserved mitochondrial function to provide proper support to WAT metabolic functions (45–47).

Concerning the expression of adipogenic transcription factors in ING and EPI fat depots, we observed that Cebpb, Cebpa, and Pparg gene expression were significantly increased in the HFD animals, but not in the HFD+n7 in the subcutaneous WAT. These findings bring us evidence that palmitoleic acid attenuates adipogenesis triggered by HFD.

In ING and EPI fat depots, the expression of inflammatory cytokines genes, Mcp1 and Tnf-α, was increased in both HFD and HFD+n7 groups when compared to control. Interestingly, the expression of these genes is more pronounced in HFD+n7 group. These findings suggest that palmitoleic acid treatment triggers changes in the immune system response to obesity in WAT. Further experiments and immunological assays must be performed. It was described that 15 days of palmitoleic acid treatment after 12 weeks of HFD promotes the reduction of TNF-α gene and protein expression in the liver of C57BL/6 mice (23), but we could not detect this alteration in our experimental protocol.

When macrophages derived from bone marrow were grown in a lipid-enriched medium mimicking the condition of animals subjected to HFD, treatment with palmitoleic acid successfully reversed TNF-α gene and protein expression of these cells (48). Besides, it was shown that palmitoleic acid promoted an increase in oxygen consumption in macrophages and in 3T3-L1 adipocyte (19). Palmitoleic acid prevented insulin resistance in the C2C12 muscle cell line, inhibiting the palmitic acid activation of macrophage J774 (22). Also, palmitoleic acid reduced inflammation of macrophage activated by lipopolysaccharide (LPS), inhibiting nuclear factor κB. It was previously shown that palmitoleic acid has a relevant anti-inflammatory effect reducing TNF-α, interleukin 6 (IL-6), and MCP1 expression by intraperitoneal activated macrophages (21).

In this work, we found increased WAT inflammation in HFD+n7 group. It is possible that 16:1n7 is exerting a counter-regulatory mechanism in WAT, because it was described that palmitoleic acid has important anti-inflammatory action in macrophages and hepatocytes (21, 23). Interestingly, genetically modified mice with reduced TNF-α signaling fed an HFD for 11 weeks showed an increase in glucose intolerance. Besides, mice with 10 days of life showed diminished WAT adipogenic capacity, as well as increased hepatic steatosis, indicating that TNF-α signaling pathway is essential for WAT expansion and remodeling during obesity (49). These data corroborate the increased Tnf-α expression found in our experimental model.

On the other hand, it was described that leptin increases TNF-α and IL-6 production by monocytes (50) and that the treatment with LPS and TNF-α induces the increase in leptin mRNA levels in hamster epididymal, omental, renal, and subcutaneous WAT (51). TNF-α also induces the increase in leptin gene expression in 3T3-L1 adipocytes (52). Therefore, the increased expression of Tnf-α supports the increased leptin levels in HFD and HFD+n7 groups in our experimental model.

Leptin is an important marker of body adipose mass and positively correlates with it (53). The subcutaneous WAT is the main leptin source (54). In agreement with that, we have detected an increase in *leptin* gene expression for both ING and EPI fat depots of HFD animals, although this increase is less pronounced in the HFD+n7 group.

Beyond these findings, the consumption of an HFD increased Nos2 gene expression in ING fat depot, but this increase was partially prevented by 16:1n7. This gene encodes for a nitric oxide–inducible enzyme [inducible nitric oxide synthase (iNOS) or NOS2] that catalyzes the production of nitric oxide. Other studies have shown that palmitoleic acid promotes a reduction of Nos2 in bone marrow macrophages of mice fed an HFD (48), besides leading to an increase in oxygen consumption and fatty acid oxidation in these macrophages, corroborating our previous work using 3T3-L1 adipocytes (19).

Finally, we have verified that HFD promoted an increase in Gpx gene expression in subcutaneous WAT. The increase in fatty acid oxidation, as well as other energy substrates in mitochondria, can lead to an increase in the production of reactive oxygen species such as superoxide anion and peroxynitrite, which originate from the nitric oxide production by the enzyme NOS. We hypothesize that this effect is compensation for the high lipid demand created by the HFD, suggesting a pathway to control the formation of free radicals, as the antioxidant enzymes (SOD, catalase, and GPX) are involved in the endogenous defense against oxidative stress (55, 56). Five weeks of HFD was sufficient to reduce the activity of the antioxidant enzymes catalase and glutathione peroxidase activity in rat liver (57). Besides, in our experimental protocol, 16:1n7 promoted an important increase of *Sod1* expression in the subcutaneous WAT. This result is in agreement with our recently published work with 3T3-L1 cell line where we demonstrated that 16:1n7 increases *Sod1* gene expression (19).

In that same work (19) we also showed that 16:1n7 increases fatty acid oxidation, lipolysis and lipogenesis, and intracellular ATP content, as well as oxygen consumption in 3T3-L1 cells, suggesting that palmitoleic acid increases the adipocytes' basal metabolic rate. Herein, using an HFD-induced obesity in animal model, the concomitant increase in fatty acid oxidation and lipogenesis in WAT suggests that the WAT from 16:1n7–treated animals is metabolically more active than that of non-treated animals, corroborating our previous published work in 3T3-L1 adipocytes.

**Figure 6:**
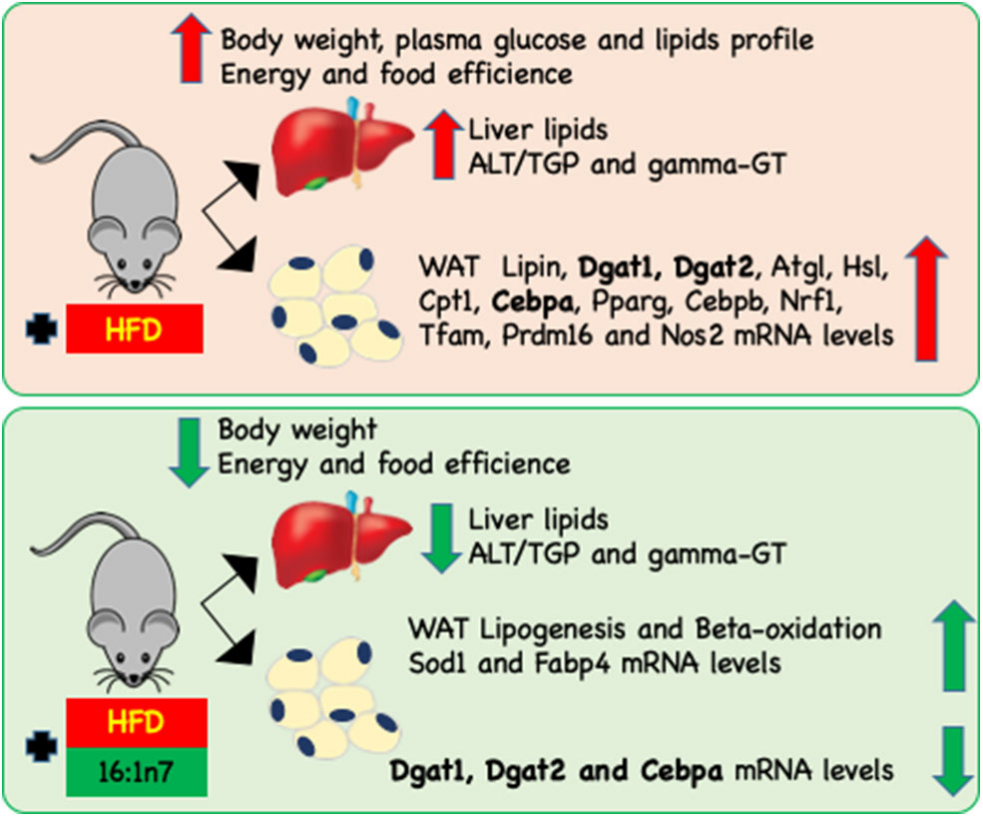
Schematic representation of changes induced by HFD on the body, liver, and inguinal (ING) adipose depot attenuated by 16:1n7 supplementation. HF diet led to increasing of body weight, food and energy efficiency, plasma glucose, triglyceride, total cholesterol and fractions, ALT/TGP and γ-GT, liver TAG, and total cholesterol levels, as well as *lipin, Dgat1, Dgat2, Atgl, Hsl, Cpt1, Cebpa, Pparg, Cebpb, Nrf1, Tfam, Prdm16*, and *Nos2* mRNA levels in ING WAT. Treatment with 16:1n7 for 28 days reversed many of these effects. Additionally, 16:1n7 increased ING adipocytes lipogenesis and β-oxidation of fatty acids, and increased *Sod1* and *Fabp4* mRNA levels.

In brief, palmitoleic acid treatment softened the body mass gain, prevented some aspects of hepatic steatosis, regulated lipogenesis and fatty acid oxidation in ING adipocytes, and modulated the expression of genes involved in the main WAT metabolic activity (lipolysis and lipogenesis), as well as genes related to mitochondrial function and biogenesis, fatty acid oxidation, adipogenesis (cell differentiation), and inflammation-related genes (Figure 6). The action of 16:1n7 is subtle but beneficial, and our data suggest that when the results caused by both HFD and palmitoleic acid are in the same direction, they add to each other, but when these effects are opposite, they are overlapped by the HFD-induced obesity (at least initially), except by gene expression changes, which we do not know if or how it will affect adipocyte metabolic activities, energy homeostasis and therefore in animal body mass in the long term.

HFD is a challenge of metabolic regulation and energy homeostasis. If HFD-induced obesity progresses, it leads to changes in WAT metabolic activity to adapt/survive to this newly imposed condition. Interestingly, animals receiving palmitoleic acid treatment seem to require fewer adjustments, softening these changes, at least concerning gene expression and hepatic parameters. Further studies will be required to elucidate these 16:1n7 effects in obesity. Our data suggest that palmitoleic acid treatment reduces the need for adaptation imposed to the organism by the HFD, which is a challenge and, in the long term, prejudicial.

## Data Availability Statement

The raw data supporting the conclusions of this article will be made available by the authors, without undue reservation, to any qualified researcher.

## Ethics Statement

The animal study was reviewed and approved by Ethics Committee on Animal Use of the Federal University of São Paulo (CEUA 8347020315).

## Author Contributions

MC, JS, RS, TF, VS, FA, VA, and LA-C performed the experiments, analyzed the results, and revised the manuscript. MA-V designed the study, analyzed the results, and supervised the study. All authors contributed to the article and approved the submitted version.

## Conflict of Interest

The authors declare that the research was conducted in the absence of any commercial or financial relationships that could be construed as a potential conflict of interest.
